# Synthetic cell division via membrane-transforming molecular assemblies

**DOI:** 10.1186/s12915-019-0665-1

**Published:** 2019-05-24

**Authors:** Simon Kretschmer, Kristina A. Ganzinger, Henri G. Franquelim, Petra Schwille

**Affiliations:** 1 Department of Cellular and Molecular Biophysics, Max-Planck-Institute of Biochemistry, Am Klopferspitz 18, 82152 Martinsried, Germany; 2 Present address: Department of Bioengineering and Therapeutic Science, University of California San Francisco, San Francisco, California, USA; 3 Physics of Cellular Interactions Group, AMOLF, Amsterdam, The Netherlands

**Keywords:** Minimal cell, Model membrane systems, In vitro reconstitution, Bottom-up synthetic biology, FtsZ, MinCDE, Actomyosin, DNA origami

## Abstract

Reproduction, i.e. the ability to produce new individuals from a parent organism, is a hallmark of living matter. Even the simplest forms of reproduction require cell division: attempts to create a designer cell therefore should include a synthetic cell division machinery. In this review, we will illustrate how nature solves this task, describing membrane remodelling processes in general and focusing on bacterial cell division in particular. We discuss recent progress made in their in vitro reconstitution, identify open challenges, and suggest how purely synthetic building blocks could provide an additional and attractive route to creating artificial cell division machineries.

## Synthetic cell division: splitting membrane compartments

Although it is difficult to conclusively define the distinct properties of living matter, it is a remarkable fact that all species of life are able to decrease their internal entropy (i.e. maintain and increase their complexity) at the expense of substances or free energy taken in from the environment [1]. Thus, in order for life to develop its characteristic complexity, the exchange of matter and energy between a living organism and its environment has to be regulated. This task has been solved by confining its molecular components to isolated compartments, the first representatives of biological cells. In order to reproduce—another distinctive feature of living systems—cells need to grow and divide into two daughter compartments. The structures that have evolved to generate chemically tight but mechanically flexible compartments (cells or organelles) are *biological membranes*. Thus, controlling large-scale membrane transformations is a prerequisite for reconstituting (proto-) cell division in minimal systems.

Membranes in “modern” cells are sheet-like structures that are mainly composed of two classes of biomolecules: lipids and proteins. The amphipathic properties of lipids make them ideally suited to separate polar environments: they can spontaneously organize into a lipid bilayer, the basic scaffold of any biological membrane. Whereas the protein components of biological membranes are not essential for the formation of this scaffold, proteins are crucial to the many biological functions of membranes: among other things, membrane proteins mediate the controlled exchange of molecules across the “barrier” created by the lipid bilayer and sense changes in the environment.

In addition to their role as active boundaries, membranes are dynamic structures, and their constituent lipids and proteins can diffuse rapidly in the plane of the membrane. Beyond 2D rearrangements, biomembranes and the underlying cortex undergo constant topological changes to fulfil their biological role: changes in membrane morphology are involved in endo- and exocytosis, cell and organism homeostasis, nutrient uptake and sensing, and cell mobility. A multitude of intracellular processes involving membrane-bound organelles also rely on the remodelling of membrane structures to maintain the organelle shape and functionality (e.g. autophagy). In endo- and exocytosis, membrane vesicles fuse or pinch off from the plasma membrane and organelles. These fission and fusion processes are also important on the scale of entire cells, underlying cell division and processes such as gamete fusion.

A major aim of the discipline of bottom-up synthetic biology is to create ‘minimal cells’—rationally designed entities whose life-like properties arise from the successful reconstitution of the fundamental cellular processes, such as an externally sustained metabolism and self-replication [2]. Such simplified model cells would not only have great potential as efficient bioreactors for industrial biotechnology, but also provide a route to answering fundamental questions about life in general: what defines life, how could it have originated from inanimate matter, and can it be, at least partially, reconstituted from defined molecular components, be they of natural or of synthetic origin?

**Fig. 1.**
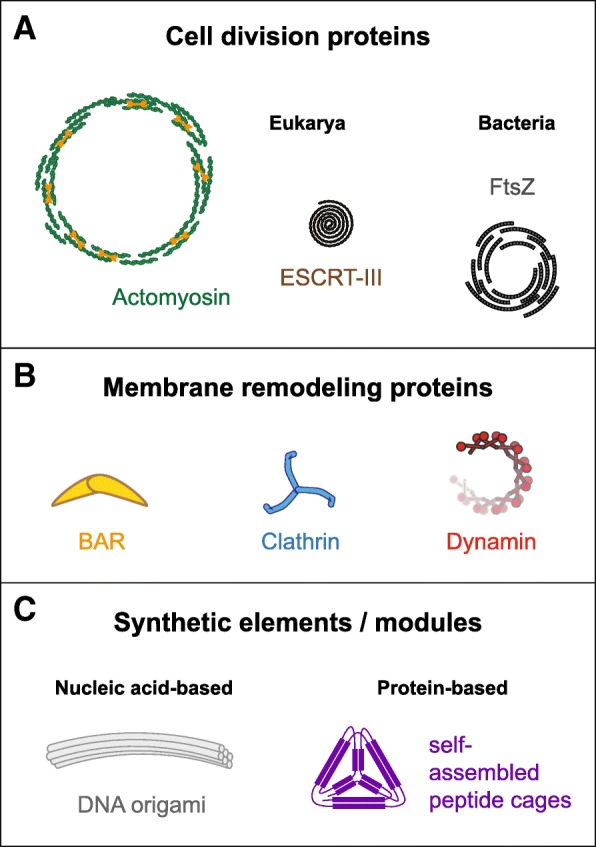
Examples of biological and synthetic membrane shaping proteins and elements. **a** Key proteins involved in membrane shaping during cytokinesis in eukaryotic cells (i.e. actomyosin and ESCRT complexes) and cell division in bacteria (i.e. FtsZ). **b** Classic membrane remodelling proteins involved in endocytosis (e.g. BAR domains, clathrin and dynamins). **c** New synthetic and shape-programmable modules (e.g. DNA origami and self-assembled peptide cages) can be employed as artificial membrane shaping elements

Given the essential role of biomembrane reshaping in cell function, it is clear that any attempt to create such a minimal cell will have to include a basic set of molecular machineries capable of mediating these membrane transformations (Fig. 1a, b). In particular, the process of cell division is a key feature of living systems that a minimal cell would need to recapitulate, as it is a fundamental prerequisite for its reproduction. In this review, we discuss how the joint work of researchers from the life sciences, as well as from the physical sciences and engineering, has been crucial for improving our mechanistic and quantitative understanding of these membrane processes. We present examples for the in vitro reconstitution of membrane transformation phenomena, focusing in particular on the reconstitution of bacterial cell division. Therefore, we also discuss the recent work on the reconstitution of positioning systems for cell division machineries. We close with a perspective on how rationally designed, artificial supramolecular machines (e.g. using DNA origami or designer proteins and peptides; Fig. 1c) could replace naturally occurring protein assemblies in mediating membrane bending, shaping and fission in artificial cells.

## Model systems for studying the biophysics of membrane transformations

The biophysics of membrane deformations has been studied for decades, both experimentally and theoretically [3–6]. Most experimental studies use one of the following three model membrane systems: supported lipid bilayers (SLBs), small or large unilamellar vesicles (SUVs, 20–80 nm; LUVs, 50–400 nm) or giant unilamellar vesicles (GUVs, > 1 μm) [7]. SUVs and LUVs are useful membrane models for studying protein–membrane interactions and, in particular, curvature recognition [8]. SLBs are a very versatile model system, typically formed by initiating the rupture and fusion of SUVs on solid substrates. While their planar nature makes them ideal for high-resolution microscopy studies (e.g. using total internal reflection fluorescence or atomic force microscopy), interactions with the support can be problematic, because the membrane fluidity is compromised and the membrane sheet cannot be deformed as it is stabilised by the solid support. The latter is in particular a limitation when studying membrane shape transformations. To some extent, these interactions may be reduced by functionalising lipids or surfaces with polymers [9], but the free-standing membranes of GUVs offer a much-used alternative model membrane system [10]. Since GUVs are cell-sized, they also emulate cell-like geometric and volumetric boundary conditions and they are sufficiently large to be imaged by optical microscopy. Most importantly for studies of membrane transformations, GUVs can be micro-manipulated because of their size, e.g. to generate membrane tubules or measure membrane tension [11].

## Protein assemblies can drive membrane remodelling

Membranes have an intrinsic tendency to bend towards one side rather than towards the other, which is characterised by the spontaneous curvature first introduced by Helfrich [12] as a key parameter for a physical continuum description of membranes. Importantly, the spontaneous membrane curvature can be affected by any particle interacting with the lipid bilayer, such as ions or proteins [13]. As long as the total membrane area remains constant, a change in spontaneous curvature will result in shape changes of the membrane [14]. Particularly large spontaneous curvatures are induced by the adsorption of amphipathic peptides [15] and (Bin/amphiphysin/Rvs) BAR domain proteins [16]. For example, the adsorption of the antimicrobial peptides temporins B and L to SLBs caused the extrusion of membrane tubules [17]. BAR proteins (Fig. 1b) have intrinsically curved shapes that, at low densities, act as curvature sensors while at high densities can also induce membrane curvature, as shown by in vitro reconstitution experiments [18–21]. While BAR domain proteins are thought to direct actin cytoskeleton remodelling (Fig. 1a) to sites of endocytosis, other protein machineries are also required for the membrane transformations during cytokinesis. In eukaryotic cells, the endosomal sorting complex required for transport (ESCRT system; Fig. 1a) fulfils this function. The molecular mechanism by which ESCRT induces membrane curvature is still debated, but in vitro experiments on SLBs have now suggested that a main component of the ESCRT-III complex self-organizes into spiral ‘springs’ that store the energy required for membrane deformation upon triggering the spring’s release [22]. Recently, the reconstitution of ESCRT-III inside GUVs has shown that forces resulting in membrane scission could be generated in nanotubes pulled from these vesicles in an ATP-dependent manner by the combined action of Snf7, Vps24, Vps2 and the Vps4 ATPase [23], solving a longstanding dispute over the involvement of Vps4 in the abscission process. While in these experiments the GUVs were made by electroformation, a novel method based on laser-induced fusion of GUVs was also recently used to reconstitute ESCRT-III proteins inside them [24], allowing more temporal control of the experimental system. It was found that CHMP2B, homologous to Vps2, may maintain synaptic spine structures by forming a diffusion barrier for lipids at membrane necks by CHMP2B polymers. In addition to dedicated division proteins, high densities of proteins engineered to interact with membranes, such as His-tagged GFP, can also induce membrane transformations and even membrane fission, irrespective of the protein’s intrinsic shape, as shown by in vitro experiments on GUVs [25, 26]. While membrane transformations such as budding and tubulation have been recreated in vitro by introducing the proteins that trigger them in nature (e.g. clathrin [27]; Fig. 1b), so far, the controlled division of phospholipid vesicles has not yet been achieved, even in the absence of stabilising structures such as the actin cortex or the bacterial cell wall. This is perhaps not entirely surprising, given that reconstitution of a controlled division site requires precise spatiotemporal control over the localization and action of the membrane-deforming protein machineries. Recent efforts towards in vitro models of cell division, as well as a discussion of the processes that underpin their biological inspiration, are the topics of the following sections.

## Towards synthetic cell division in vitro

**Fig. 2.**
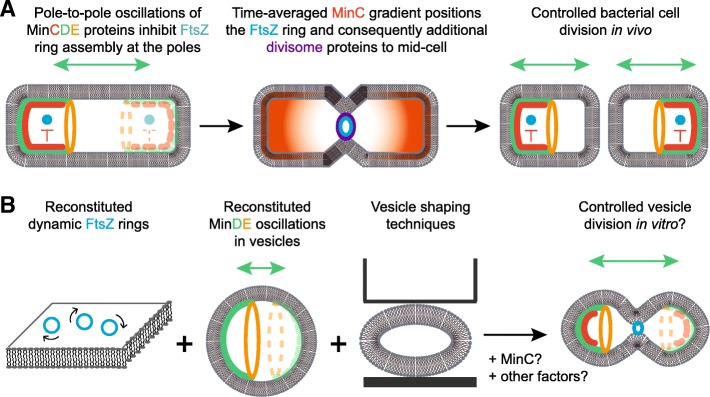
Cell division in vivo and potential reconstitution in vitro. **a** Simplified depiction of FtsZ and divisome localization by the MinDE-dependent MinC gradient in *E. coli*. Components of the nucleoid occlusion mechanism, FtsZ-anchoring proteins, the cell wall and other factors discussed in the text are omitted in this scheme for clarity. **b** Conceptual depiction of a potential realization of synthetic vesicle division based on *E. coli* division proteins

Reconstituting cell division in vitro represents a desirable, albeit ambitious, goal towards realizing the bottom-up construction of an artificial cell. In biological systems, cell division involves the segregation of chromosomes, organelles and other intracellular components, and cytokinesis, the physical splitting of the cell envelope. In light of this review’s focus on membrane transformations, we focus exclusively on cytokinesis (Fig. 2a) and its reconstitution. Cytokinesis is orchestrated by the “divisome”, a species-specific set of cytoplasmic and membrane-bound proteins that together constitute the required molecular machinery for constricting and splitting the mother cell envelope [28].

In addition to these membrane-transforming processes, an important aspect of cytokinesis is its spatiotemporal regulation. In order to divide at the right time and location, cells have evolved both positive and negative regulatory mechanisms to control divisome assembly [29]. While positive regulatory systems recruit and/or stabilize divisome proteins at the division site, negative regulatory mechanisms inhibit division at sites of unwanted division.

A reconstitution of divisome elements from different kingdoms of life [30] (Fig. 1a) has been separately attempted for the actomyosin-based contractile machinery [31] and ESRCT system [32] of eukaryotic cells, the bacterial machinery based on FtsZ [33–36] and the ESRCT-like Cdv machinery of archaea [37]. In the following, we focus on bacterial divisome elements, for which we summarize relevant work regarding their in vitro reconstitution. We then discuss recent progress towards the de novo design of membrane-transforming and divisome-positioning elements.

Although we focus on cell division driven by specific membrane-transforming elements at the division site, it is important to note that cytokinesis can also occur without such machineries. A prominent example is presented by L-forms, bacterial variants lacking a cell wall that can be generated for both Gram-positive and Gram-negative bacteria [38]. Even in the absence of the highly conserved protein FtsZ, L-form bacteria have been shown to divide by biophysical mechanisms involving excess membrane synthesis coupled to cell shape changes [38, 39]. Moreover, certain bacteria, including *Mycoplasma genitalium*, divide via motility of the nascent daughter cells on solid surfaces, when FtsZ is deleted [40]. The Szostak lab has worked extensively on protocell model systems using vesicles that self-assemble from fatty acid micelles [41]. They could show that in a solution where solute permeation across the membranes is slow, modest shear forces introduced by blowing puffs of air onto the sample from a distance were then sufficient to cause the vesicles to divide into multiple daughter vesicles without content loss [41, 42]. It is plausible that processes resulting in similar fluid shear stresses might have occurred on the early Earth, pointing to a potential avenue for simple, physical division mechanisms employed by primitive cells. Moreover, their further study may provide principles that can be employed to realize similar mechanisms in the context of synthetic cells and their division.

## Bacterial cell division

Bacterial cytokinesis is a complex dynamic process that involves the synthesis of new cell envelope material, membrane constriction and fission as well as remodelling and separation of the peptidoglycan layer [43]. Cell division in the vast majority of bacteria involves the GTPase protein and tubulin homologue FtsZ [43]. FtsZ (Figs. 1a and 2a) polymerizes into a dynamic ring-like structure at the division site, referred to as the “Z-ring” [44], where it is anchored to the membrane by the adaptor proteins FtsA and ZipA [45, 46]. Together, these three proteins comprise the “proto-ring”, which serves to recruit further divisome proteins [47]. Importantly, the FtsZ ring is not a uniform, cohesive structure, but comprised of smaller, overlapping filaments [48]. These filaments are highly dynamic and exhibit treadmilling behaviour [33, 49, 50]. Interestingly, FtsZ treadmilling is coupled to circumferential movement of the cell wall synthesis machinery in the periplasm [49, 50], although the molecular mechanism of this coupled motion remains unclear [51]. Moreover, in *Escherichia coli*, cell wall synthesis and not FtsZ limits the rate of constriction [52]. Thus, it has been suggested that FtsZ has mostly an organizing function and that it is the cell wall synthesis machinery which generates constrictive force via the pushing of newly inserted peptidoglycan against the inner membrane from the periplasm [43]. However, in vitro reconstitution experiments have suggested that FtsZ actively generates forces capable of membrane remodelling [34]. Thus, the individual contributions of FtsZ and cell wall synthesis are interesting open questions and motivate further research in this area [51].

Mechanisms for the localization of FtsZ to the division site differ between bacteria, and both positive and negative regulatory mechanisms have been reported [29]. In *E. coli*, two negative regulatory systems synergistically allow for FtsZ polymerization exclusively at the mid-cell plane: (1) the nucleoid occlusion mechanism, and (2) the MinCDE system. The first involves the protein SlmA and inhibits Z-ring assembly across the chromosome [53]. The MinCDE system inhibits assembly near the poles via a self-organized gradient of the FtsZ inhibitor MinC, which has the highest concentration at the poles and lowest at the mid-cell [54, 55]. This gradient is generated by pole-to-pole oscillations of the peripheral membrane-binding ATPase MinD and its ATPase-activating protein MinE, which MinC follows as a passenger [56, 57]. Importantly, pole-to-pole oscillations, and consequently correct gradient formation, arise from a sensitive interplay of geometric boundary conditions and other parameters, such as interaction rates [58, 59]. Out of those two positioning systems, the *E. coli* Min system, including functional gradients, has been reconstituted in various in vitro environments, as will be discussed below.

## Synthetic cell division via reconstitution of *E. coli* divisome elements in vitro

In a simplified system, controlled division of a lipid vesicle should involve at least a biomolecular assembly capable of membrane transformation as well as a mechanism that positions it to the middle of the vesicle. The corresponding machinery in *E. coli*, namely FtsZ and the Min system, appear promising in this regard due to the low number of involved, and relatively well characterized, components. However, due to the sensitivity of the Min system to the geometry and dimensions of the surrounding membrane system [59–62], the vesicle will likely need to be shaped in a way to enable robust gradient formation, and FtsZ localization, by the Min system.

Towards reconstituting vesicle division based on *E. coli* proteins, substantial progress has been made both for the reconstitution of FtsZ and the Min system on model membranes. In the following, we briefly summarize the outcomes of reconstitution experiments with these components, which are reviewed in more detail elsewhere [63–66]. With regard to FtsZ, several reconstitution studies relied on a fusion protein (FtsZ-YFP-MTS), in which FtsZ is C-terminally truncated and linked to a fluorescent reporter followed by the amphipathic membrane targeting sequence (MTS) of MinD [34, 36, 67]. Conveniently, this chimeric protein can bind to lipid membranes in the absence of FtsZ’s natural anchor proteins FtsA and ZipA [34], thus simplifying reconstitution experiments. When reconstituted inside multilamellar liposomes, FtsZ-YFP-MTS was capable of membrane deformation [34], although it is unclear whether this force would suffice for constriction in vivo [43]. Furthermore, FtsZ-YFP-MTS was found to display an intrinsic curvature in its polymeric state, facilitating its self-assembly along membranes of negative curvature [67]. On supported lipid bilayers, FtsZ self-organizes into dynamic ring structures, in which individual FtsZ filaments undergo treadmilling to drive chiral rotations of the rings [33] (Fig. 2b). Initially, it has been suggested that formation of these dynamic rings requires the simultaneous presence of (non-MTS-fused) FtsZ and the anchor protein FtsA, which exerts a negative feedback on membrane-bound FtsZ filaments [33]. However, a subsequent study from our lab demonstrated that, under certain biochemical conditions, FtsZ-YFP-MTS alone also self-organizes into dynamic ring patterns [36]. Importantly, one decisive factor determining the type of emerging pattern (rings or filamentous structures), was found to be the concentration of free Mg^2+^ [36]. This result has important implications for Z-ring formation within the context of synthetic cell division, as complexity can now be reduced to a single chimeric protein and because the required conditions for correct assembly are better defined. Very recently, it has been found by in vitro reconstitution that the essential divisome proteins FtsN and FtsQ co-migrate with treadmilling FtsZ filaments via a diffusion-capture mechanism [68].

FtsZ variants have also been reconstituted inside lipid droplets [69], coacervates [70], crowding-induced phase-separated condensates [71] and lipid vesicles [35, 72, 73]. Besides the already mentioned deformations observed for FtsZ-YFP-MTS in multilamellar vesicles [34], the simultaneous presence of FtsZ and different ZipA or FtsA variants has been reported to give rise to membrane deformations when reconstituted or expressed inside giant unilamellar vesicles [35, 72, 73]. In some cases, these deformations have been suggested to be responsible for observed constriction and division of vesicles [35].

Among different positioning systems, the Min oscillator is a promising option for localizing an FtsZ-based divisome in the middle of a vesicle in vitro as it contains only a few, relatively well-understood components and the influence of biochemical and geometrical factors has been comprehensively analyzed. When MinD and MinE are reconstituted on a flat supported membrane, topped by a uniform buffer, these proteins self-organize into traveling waves via an ATP-driven reaction-diffusion mechanism [74]. Such simplified flat membrane systems have been used extensively by us and others to investigate the effects of lipid and buffer composition, as well as mutations in MinD and MinE, on the formation and properties of Min patterns [75–79], and to achieve external (photo-)control over self-organization [80]. Moreover, additional division-related proteins, including MinC, FtsZ and ZipA variants, have been added to the reconstituted MinDE patterns [81–83]. Although the experiments above were performed in the presence of a two-dimensional, non-enclosed membrane system, the simplicity of the setup allowed the efficient establishment of suitable conditions for the functionality and compatibility of different components as well as the potential to modulate the spatiotemporal properties of Min patterns in a predictable fashion.

We and others have also reconstituted Min protein patterns in more cell-like settings, such as in PDMS microcompartments [60, 61, 84], in lipid droplets [85], on the outside of lipid vesicles [86], and—most recently and relevant for this review—inside lipid vesicles [87] (Fig. 2b). These studies established which types of patterns form under different geometric constraints and, with regard to the reconstitution in droplets and vesicles, confirmed that Min oscillations can occur inside lipid-mono- or -bilayer-enclosed compartments. Intriguingly, Min dynamics in lipid vesicles resulted in shape changes in concert with the oscillations, resulting in an apparent, periodic “beating” of the vesicles [87]. Potential roles of these mechanical effects in cell division could be explored in future studies.

Notably, the Min system has also been combined with additional division-related proteins in some of the above-mentioned cell-like systems. In lipid droplets, Min proteins and FtsZ-YFP-MTS oscillated in an anti-correlated manner [85]. Moreover, oscillations of Min proteins in PDMS microcompartments resulted in a time-averaged concentration gradient of MinC with maxima at the poles and minimum in the middle [61]. This gradient was capable of localizing FtsZ-YFP-MTS filaments to the middle of the compartment [61]. Very recently, our lab has shown that—even in the absence of MinC—MinD and MinE can support the anticorrelated movement and oscillation of model membrane proteins, including mCherry fused to various membrane targeting sequences, lipid-anchored streptavidin and FtsZ-YFP-MTS [88]. Moreover, if the proteins are permanently anchored to the membrane, MinDE oscillations can localize them to the middle of a microcompartment [88]. This implies that MinD and MinE are sufficient to generate a generic cue for the localization of membrane proteins, which may also be relevant for simplified divisome localization machineries.

## Challenges for the in vitro reconstitution of divisome elements

Despite the progress in reconstituting bacterial divisome elements in vitro, several challenges remain to be addressed. First, it has still not been experimentally demonstrated that FtsZ can reproducibly exert sufficient forces to constrict and divide a lipid vesicle from the inside. Quantitative measurements of potential forces generated by FtsZ could resolve its sufficiency or contribution for vesicle division. Second, while FtsZ forms dynamic rings and the Min system is capable of gradient-forming pole-to-pole oscillations in vitro, the integration of both phenomena is not as trivial as may seem. While the reconstituted FtsZ(−YFP-MTS) rings are of similar spatial dimensions to the Z-rings observed in vivo, the reconstituted Min patterns, oscillations and gradients are roughly one order of magnitude larger than the ones occurring in vivo, for reasons that are still not fully understood. Although several factors, like lipid composition, crowding agents and the concentration and functional features of Min proteins have been identified that modulate the length scale of Min patterns [60, 75, 76, 78], Min oscillations have not yet been realized in a cell-sized compartment, but rather in compartments scaled to the dimensions of in vitro Min patterns [60, 84], which are around an order of magnitude larger than the in vivo patterns [74]. Additionally, robust vesicle division is likely to require the vesicles to assume a rod-like shape to stabilize gradient-forming Min oscillations and adjust the vesicle’s curvature for Z-ring assembly along the inner vesicle circumference. Microfabrication approaches to sculpt vesicles into a defined shape (Fig. 2b) appear as a promising strategy in this regard, e.g. 3D printed protein cages that can change shape with pH, or squeezing GUVs into shape-imposing microfluidic (PDMS) traps. Lastly, it will be interesting to test if a membrane-targeted FtsZ variant, MinD and MinE are indeed the only necessary protein components for controlled vesicle division, or if MinC and potentially other factors are required. Reconstitution attempts with and without additional components are expected to produce new and interesting insights into the detailed roles of the tested factors.

## Synthetic cell division based on non-natural biomolecular components

As discussed in the previous sections, synthetic cell division could be achieved by reconstituting well-understood proteins derived from living systems in a cell-free setting (Fig. 2). More radically, and complementary to the reconstitution of natural divisome elements, novel division machineries could be engineered that are inspired by nature and/or devised from scratch. While such elements often share little resemblance in sequence or even their constituent material with natural proteins, they may nonetheless be inspired by, or based on properties abstracted from, their natural counterparts. Alternatively, they can be built from first principles, which appears more attainable now than previously, due to progress in protein engineering [89]. Although the use of designed molecules does not necessarily reveal how living systems divide, it can reveal core principles of a biologically relevant phenomenon, in our case the controlled division of a membrane-enclosed system. Moreover, the possibility of tailoring designs for a specific experimental purpose may also facilitate the programmable variation of their inherent biochemical parameters.

A prominent example for a programmable nanometre-scale building material that has shown considerable success with regard to membrane transformation is DNA origami (Fig. 1c). In this methodology, DNA’s specific base-pairing and self-assembly properties are exploited to use it as a structural material to generate objects of pre-designed shapes [90]. Taking advantage of this programmability, DNA origami has successfully been employed to achieve membrane binding [91, 92] and transformation [93, 94]. For example, we have shown that variably curved DNA origami objects mimicking banana-shaped BAR domains, targeted to membranes via cholesterol anchors, can recognize and deform GUVs [94]. These DNA origami objects recapitulated structural and functional properties of natural BAR domains (Fig. 1b), including membrane curvature generation [94]. Analogously, in a recent study, it has been shown that polymerizing DNA origami curls, inspired by dynamin (Fig. 1b) and ESCRT (Fig. 1a) proteins, could also tubulate membranes [95]. Moreover, DNA origami cages and rings have been employed to template various shapes of lipid vesicles [96–99]. These studies demonstrate the potential of DNA origami to transform lipid membranes, which, upon further design and modification, could potentially also enable controlled vesicle division.

Besides DNA origami, engineered peptides or proteins could also serve as artificial membrane-transforming elements. While still not as advanced as DNA origami in terms of programming arbitrary shapes, the de novo design of protein structure and function has progressed dramatically in recent years [89, 100]. For example, the engineering of artificial peptide and protein cages (Fig. 1c) and similar assemblies indicates a diminishing gap between the capabilities of DNA- and protein-based molecular design [101–103]. Considering the chemical diversity of natural and unnatural amino acids in proteins that contrasts with inevitably high negative charge of DNA origami, it is likely that protein design will play an important role in future efforts to create an artificial cell, potentially also with applications in artificial membrane transformation and division. An important challenge with respect to both artificial protein- and DNA-based machineries will be the realization of dynamic behaviour. Dynamics are typically required for membrane transformation and based on consumption of chemical energy, as illustrated by the highly dynamic division components found in living systems, including FtsZ and actomyosin [31, 33].

## Promises and frontiers associated with synthetic cell division

**Fig. 3.**
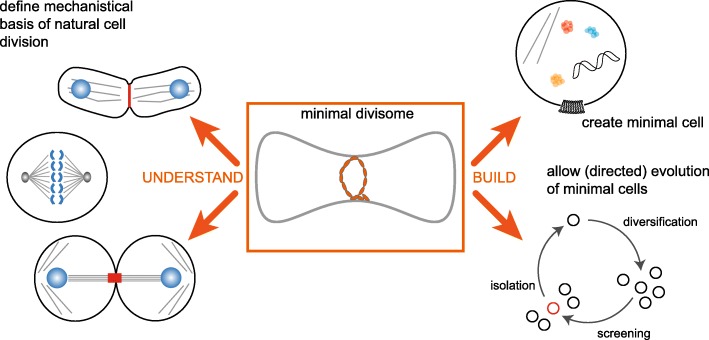
Relevance and potential applications of defining and creating a synthetic cell division machinery. A minimal model system that can recapitulate cell division will be useful to understand the mechanistic basis of the process in cells, in particular by defining the elements that are both necessary and sufficient to achieve division (*left side*). A minimal cell will need to be capable of dividing to mimic one of the essential characteristics of life (*right side*, *top*) functionalities. Once DNA or RNA replication can be successfully reconstituted in a minimal cell, both growth and division would be required to evolve these minimal cells, for example by cycles of error prone duplication of the genetic material followed by selection of a desired functionality (*right side*, *bottom*)

Clearly, we are only at the beginning of reconstituting controlled large-scale membrane transformations as required for (proto-) cell division in minimal systems. However, elucidating and fully recapitulating the fundamental mechanistic aspects of membrane transformations would without doubt impact on a wide range of disciplines from biology to medicine, given that cell division is constitutive of processes from embryo development to cancer (Fig. 3). Reconstitution of cell division from the bottom up could also unravel the similarities and differences between the division machineries of different kingdoms of life. Finding the “smallest common denominator” or common motif between the many solutions found by evolution could be crucial for the design of a synthetic minimal division machinery that may well combine elements from more than one species. Beyond using, or repurposing, natural routes, the synthetic biologist’s toolbox will be expanded by the use of artificial nanomachines, such as DNA-based constructs or synthetic designer peptides and proteins. If minimal cell division were implemented together with a positioning system, the symmetry of minimal cell division could be controlled, for example by exploiting the MinDE system or the Rho-family small GTPase Cdc42 and its corresponding GAPs or GEFs [104]. Regardless of the precise implementation, reconstitution of a minimal division machinery will increase the versatility of synthetic cells and pave the way towards their directed evolution—something that to-date has not yet been successfully demonstrated for any reconstituted, man-made entity or proto-cell (Fig. 3). This would not only open up entirely new avenues for bottom-up synthetic biology, but also point to how life could have emerged from inanimate matter and provoke us to revisit our current definition of cellular life.
